# Targeted DNA methylation of neurodegenerative disease genes via homology directed repair

**DOI:** 10.1093/nar/gkz979

**Published:** 2019-11-04

**Authors:** Christopher P Cali, Daniel S Park, Edward B Lee

**Affiliations:** Translational Neuropathology Research Laboratory, Department of Pathology and Laboratory Medicine, University of Pennsylvania, Philadelphia, PA, USA

## Abstract

DNA methyltransferases (DNMTs) are thought to be involved in the cellular response to DNA damage, thus linking DNA repair mechanisms with DNA methylation. In this study we present Homology Assisted Repair Dependent Epigenetic eNgineering (HARDEN), a novel method of targeted DNA methylation that utilizes endogenous DNA double strand break repair pathways. This method allows for stable targeted DNA methylation through the process of homology directed repair (HDR) via an *in vitro* methylated exogenous repair template. We demonstrate that HARDEN can be applied to the neurodegenerative disease genes *C9orf72* and *APP*, and methylation can be induced via HDR with both single and double stranded methylated repair templates. HARDEN allows for higher targeted DNA methylation levels than a dCas9-DNMT3a fusion protein construct at *C9orf72*, and genome-wide methylation analysis reveals no significant off-target methylation changes when inducing methylation via HARDEN, whereas the dCas9-DNMT3a fusion construct causes global off-target methylation. HARDEN is applied to generate a patient derived iPSC model of amyotrophic lateral sclerosis and frontotemporal dementia (ALS/FTD) that recapitulates DNA methylation patterns seen in patients, demonstrating that DNA methylation of the 5′ regulatory region directly reduces *C9orf72* expression and increases histone H3K9 tri-methylation levels.

## INTRODUCTION

Methylation of cytosine is a chemical modification of DNA associated with a myriad of regulatory processes in the mammalian genome such as regulation of gene expression, stabilization of repeat elements, X-inactivation and maintenance of genomic integrity (1). Alterations in DNA methylation are associated with numerous diseases, including cancer and neurodegenerative diseases such as those caused by expansion of microsatellite repeat elements (2). Aberrant methylation of the *C9orf72* promoter region has been observed in repeat expansion carriers with amyotrophic lateral sclerosis (ALS) and frontotemporal dementia (FTD) (3–5). In these patients, neurodegeneration is caused by a repeat expansion in the first intron of *C9orf72* which has been proposed to cause production of toxic RNA and protein pathologies in patient tissue (6), patient derived cells (7–10) and animal models (11–13). Interestingly, hypermethylation of the promoter occurs in ∼1/3 of patients and correlates with increased survival time, reduced neuronal loss and better verbal recall ability in FTD patients (5,14). Thus, we hypothesize that hypermethylation in the context of *C9orf72* repeat expansions is neuroprotective. However, to define causality between hypermethylation and disease pathology, there is a need to generate isogenic patient derived cell lines that accurately model this epigenetic aspect of ALS/FTD.

Current tools for targeted DNA methylation, such as the dead Cas9 (dCas9)–DNA methyltransferase (DNMT) fusion protein (15), have several features that limit their utility for generation of epigenetic disease models. Recent studies have shown that the dCas9-DNMT3a fusion protein constructs have numerous off-target effects (16–20), which may confound experiments or limit their use as therapeutic agents. Another limitation with available tools is that these constructs are usually large and difficult to transfect into patient derived primary cells or deliver via gene therapy vectors for therapeutic purposes (21), and they often require multiple gRNAs to achieve robust activity (15,16,19,22,23). Additionally, DNA methylation changes made via the fusion protein constructs are often not maintained over numerous cell divisions at some loci (15,18,24,25), making it difficult to generate stably edited cell lines for disease modeling.

Due to these limitations, we sought to develop a novel method for epigenetic editing that has fewer off target effects and enables generation of cellular disease models. While current tools rely on over-expression of synthetic DNMTs, we instead asked whether DNA methylation editing could be achieved using the endogenous double stranded DNA break repair pathways coupled with endogenous DNMTs. Upon double strand break, DNA is generally repaired either through the process of non-homologous end joining (NHEJ) or homology directed repair (HDR) (26). NHEJ involves recognition of the DNA break, generation of overhangs and finally ligation and fill-in by DNA polymerase (26). The process of NHEJ is error-prone, often resulting in insertions or deletions (indels) at the repaired site (26). Alternatively, when a repair template is present, the break can be repaired using HDR, which is typically error free but occurs at lower frequency than NHEJ (27). HDR is a complex process that involves resection of the broken DNA ends, invasion of the repair template and DNA synthesis using the repair template followed by ligation to complete the newly copied DNA (28).

We hypothesized that DNA methylation could be targeted via the process of HDR by providing the cells with an *in vitro* generated repair template containing the methylation marks to be copied into the genome. In theory, this mechanism would be similar to how DNA methylation is maintained during DNA replication. Maintenance methylation during DNA replication involves synthesis of a new DNA strand using the parental strand as a template, followed by copying of DNA methylation from the parental strand to the newly synthesized daughter strand (29). This hemi-methylated DNA recruits UHRF1 followed by DNMT1 to deposit methylation on the remaining strand, thus creating a methylation pattern that matches the original parental DNA (29–31). Here, we provide *in vitro* methylated templates to be used instead of the endogenous parental DNA template. To our knowledge, no previous study has shown that exogenous DNA methylation marks can be copied into an endogenous gene. Endogenous DNMTs, however, are known to be rapidly recruited to double stranded DNA (dsDNA) breaks (32–36). Previous studies have demonstrated that dsDNA breaks in exogenous reporter DNA constructs cause a subset of cells to gain DNA methylation of the construct, leading to gene silencing (33,36–38). Whether this acquired DNA methylation following break repair is unique to exogenously expressed DNA or also occurs at endogenous loci is unknown.

This study demonstrates that double stranded DNA breaks followed by repair via HDR with a methylated template can be used as a method for targeted DNA methylation. This novel method for editing DNA methylation takes advantage of the endogenous cellular repair machinery and does not require over-expression of a methyltransferase enzyme. We term this method Homology Assisted-Repair Dependent Epigenetic eNgineering (HARDEN) because stable methylation editing is only achieved upon DNA repair via HDR. We apply HARDEN to two genes involved in neurodegenerative diseases and find that this method exhibits vastly fewer off-target effects than a dCas9-DNMT3a fusion construct and enables generation of stably methylated cell lines that model epigenetic alterations seen in neurodegenerative disease.

## MATERIALS AND METHODS

### Plasmid and repair template generation

CRISPR gRNAs were designed using Benchling software (http://benchling.com/) and cloned into pSpCas9(BB)-2A-Puro (PX459) V2.0 vector (Addgene, Cambridge, MA, USA; Plasmid #62988)) for HARDEN experiments or pdCas9-DNMT3A-PuroR (ANV) (Addgene Plasmid #71684) and pdCas9-DNMT3A-PuroR_v2 (Addgene Plasmid #74407) as previously described (39). Single stranded oligonucleotide templates including methylated oligonucleotides were synthesized by Integrated DNA Technologies, Inc. (Coralville, IA, USA). The non-homologous oligonucleotide template is homologous against the unrelated gene *EMX1*. Sequences of gRNAs and templates are provided in Supplemental Tables S1 and S2. Double stranded DNA templates homologous to *C9orf72* were made by PCR amplification of the target region using Q5 High Fidelity polymerase (New England Biolabs, Ipswich, MA, USA) according to the manufacturer's protocol. 250 ng of purified PCR products were A-tailed in a reaction containing 0.25 mM dATP, 1.5 mM MgCl_2_, 1× Buffer II and 5 U Amplitaq polymerase (Applied Biosystems, Foster City, CA, USA) for 30 min at 70°C. A-tailed PCR products were ligated into pGEM-T Easy vector (Promega, Madison, WI, USA) and transformed into NEB Turbo competent cells (New England Biolabs) according to the manufacturer's protocol. For introduction of PAM mutations, site directed mutagenesis was carried out using the QuickChange XL kit according to the manufacturer's protocol (Agilent Technologies, Santa Clara, CA, USA). *APP* dsDNA template was synthesized and cloned into pUC-57 vector by GenScript (Piscataway, NJ, USA). Non-homologous dsDNA templates consisted of the pGEM-T Easy vector without the *C9orf72* homologous region. For methylation of plasmid repair templates, 20 μg of plasmid DNA was methylated in a 100 μl reaction containing 50 U of M.SssI CpG Methyltransferase (New England Biolabs), 640 μM SAM, 50 mM NaCl, 10 mM Tris–HCl (pH 7.9) and 10 mM EDTA for 4 h at 37°C. Enzyme was inactivated for 20 min at 65°C and then plasmid DNA was purified via phenol chloroform extraction followed by ethanol precipitation.

### Cell culture and transfection

HEK293T cells were grown in DMEM high glucose + L-glutamine (Invitrogen, Carlsbad, CA) supplemented with 10% fetal bovine serum (Atlanta Biologicals, Flowery Branch, GA) and 1× Penicillin/Streptomycin (Invitrogen). For transfection of CRISPR components, 1 μg Cas9/gRNA containing plasmid and 1 μg repair template were co-transfected into 12-well dishes using Fugene 6 (Promega) or Lipofectamine 3000 (Invitrogen) with a 3:1 transfection to DNA ratio for 48 h. For siRNA experiments, cells were transfected with 40 nM siRNA using Lipofectamine 3000 (Invitrogen). For time points longer than 2 days, puromycin selection was carried out at 1μg/ml for 3–4 additional days. For clonal line isolation, cells were diluted to 1 cell/well in 96-well plates 7 days post transfection. DNA from clonal lines was isolated using QuickExtract Solution (Lucigen, Middleton, WI, USA) according to manufacturer's protocol and screened for repair with the template using PCR and restriction enzyme digest. Induced pluripotent cell line CS52iALS-n6A was obtained from Cedars Sinai and maintained in mTeSR1 media (Stem Cell Technologies, Vancouver, Canada) and grown on Matrigel matrix (Corning, Corning, NY, USA) coated plates as previously described (7). For transfection of CRISPR components, 750 ng Cas9/gRNA plasmid + 750 ng repair template was co-transfected into 12-well dishes using Viafect reagent (Promega) with a 4:1 transfection to DNA ratio for 48 h. 0.5 μg/ml puromycin was used for selection of transfected iPSCs.

### Methylation sensitive restriction enzyme digest qPCR

Methylation sensitive restriction enzyme digest quantitative PCR (qPCR) was carried out as previously described (4). Genomic DNA was extracted using DNeasy Extraction kit (Qiagen) and 100 ng was digested with the restriction enzyme HhaI (cut site: GCGC; 1 site in amplicon). qPCR was carried out using FastStart Universal SYBR Green mastermix (Roche, Basel, Switzerland) on a StepOne Plus Real-Time PCR Machine (Life Technologies) using standard cycling. For measurement of methylation in samples transfected with 1.4 kb double-stranded templates, a forward primer was used that anneals outside the repair template and the qPCR cycling conditions were modified (38 cycles, annealing temperature 61.5°C, 1 min extension at 72°C) to increase specificity. % methylation was calculated using the formula 2^(Ct Mock – Ct Digest)^. Fold change over controls was calculated for long amplicon qPCR because percent digest verses % methylation is not 1:1. See Supplemental Table S1 for primers.

### HDR assay via PCR and restriction enzyme digest

40 ng of genomic DNA from CRISPR edited cells was amplified with 0.5 U Q5 hotstart polymerase (New England Biolabs) in a reaction containing 0.2 mM dNTP, 1× Q5 reaction buffer, 1× Q5 High GC enhancer and 0.25 μM each primer (See Supplemental Table S1 for primers). Copying of the 2 bp PAM mutation via HDR from the repair template creates a novel SspI restriction enzyme site in the amplicon. 12 ul of PCR product was digested with 8U SspI-HF (New England Biolabs) in 1X Cutsmart buffer for 2 h at 37°C. Digest products were run on either 2% agarose gels or 8% polyacrylamide gels for HDR assay and stained with SYBR Gold (Invitrogen). Bands were quantified using GelAnalyzer 2010a software (http://www.gelanalyzer.com/index.html) using valley to valley background subtraction. For the restriction enzyme HDR assay: % digest = (sum digested products)/(undigested + digested products).

### Bisulfite cloning

1 μg of genomic DNA was bisulfite converted and cleaned up using the EpiTect Bisulfite Kit (Qiagen) according to manufacturer's protocol. Bisulfite converted DNA (7.5% of total per reaction) was then PCR amplified using KAPA HiFi Uracil+ Readymix (Roche) according to manufacturer's protocol. PCR products were purified with Ampure XP beads (Beckman Coulter), A-tailed with Amplitaq polymerase (Applied Biosystems), ligated into pGEM Easy T vector (Promega) and transformed into NEB Turbo competent cells (New England Biolabs) as described above. Amplicons were sequenced at the University of Pennsylvania Sanger Sequencing core using the T7 promoter/primer using an ABI 3730 (Applied Biosystems). Bisulfite analysis was carried out using BISMA software (40) and only sequences with >90% conversion rate were included in the analysis.

### Genome wide methylation analysis

Genome wide methylation analysis was carried out using the EPIC Array (Illumina, San Diego, CA, USA), which measures CpG methylation at ∼800,000 sites. Samples were transfected for 48 h with either dCas9-DNMT3a-Puro, dCas9-DNMT3a-ANV-Puro or Cas9-Puro + 190 bp methylated template and gRNA targeting *C9orf72*. Samples were puromycin selected for 48 hours and genomic DNA was extracted 4 days post transfection. Samples were then bisulfite converted and processed using the Infinium Methylation EPIC Kit (illumina) by the Center for Applied Genomics at Children's Hospital of Philadelphia. Data was analyzed in R using the Bioconductor package ChAMP (41). Data was normalized with the beta-mixture quantile normalization method (BMIQ) and significantly differentially methylated probes were called using default parameters with the Benjamini–Hochberg procedure for false discovery rate adjustment.

### RNA expression from iPSCs

Cells were harvested via Accutase (Stem Cell Technologies), pelleted, washed in PBS and resuspended in 350 ul RLT buffer (Qiagen). Lysates were homogenized via pipetting and vortexing and RNA was extracted using the RNeasy kit (Qiagen) with on-column DNAse digest for 15 min at room temperature. 1 μg of RNA was converted to cDNA using the High-Capacity cDNA Reverse Transcription Kit (Applied Biosystems) according to manufacturer's protocol. cDNA was diluted tenfold and 4 μl was used in a qPCR reaction with 2X FastStart Universial SYBR Green master mix (Roche) and 0.25 μM of each primer. qPCR was run with default cycling conditions on a StepOne Plus qPCR machine (Applied Biosystems). Relative expression was calculated using the delta delta Ct method by normalizing to housekeeping genes (*ACTB*, *GPS*).

### Chromatin immunoprecipitation

Briefly, edited iPSC clonal lines were cross-linked with 1% formaldehyde (37°C, 10 min) and quenched with 125 μM glycine. Cells were lysed (0.5% SDS, 10 mM EDTA, 50 mM Tris/HCl, pH 8) and sonicated on high power for 30 cycles using a Bioruptor 300 (Diagenode, Denville, NJ, USA). 1 μg of chromatin was incubated overnight at 4°C with Protein A Dynabeads (Life Technologies) and 5 μl of antibody against H3K9me3 (Cell Signaling #13969) or IgG. Beads were washed with buffers of increasing stringency (low salt, high salt, lithium chloride and 1× TE buffer) and DNA was eluted in buffer containing 1% SDS. RNA and protein were digested using RNAse A (Thermo Scientific) and proteinase K (Promega). DNA was purified using PCR purification kit (Qiagen) and eluted in 200 μl EB Buffer (Qiagen). 4 μl purified DNA was inputted into a qPCR reaction as described above and percentage of input was calculated for each sample. See Supplemental Methods for extended description.

## RESULTS

### Targeted DNA methylation using oligonucleotide repair templates

**Figure 1.**
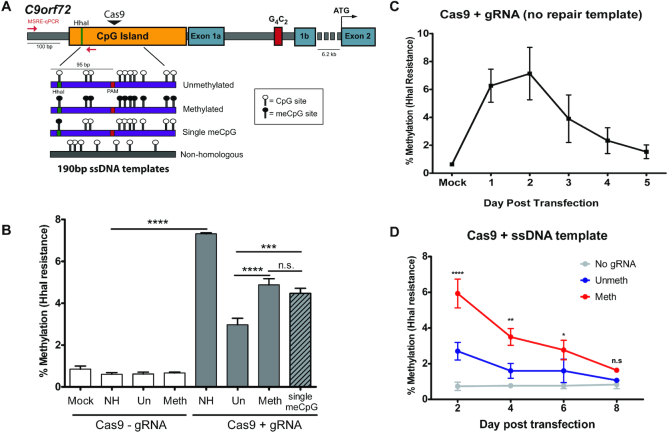
Editing DNA methylation of *C9orf72* using single stranded DNA templates. (**A**) Diagram of *C9orf72* gene structure. Red arrows indicate primers used for MSRE-qPCR. The forward primer anneals outside of the repair template. Green line indicates HhaI site that is being tested for methylation in (B). Figure drawn to scale. meCpG = methylated cytosine. (**B**) MSRE-qPCR of a CpG site within the *C9orf72* promoter in HEK293T cells transfected with CRISPR/cas9 plasmid and the indicated single stranded DNA (ssDNA) repair templates. Cells were collected 2 days post transfection. *n* = 3 (Mock, Un-gRNA, Meth-gRNA, NH+gRNA), *n* = 9 (NH-gRNA, Un+gRNA, Meth+gRNA) or *n* = 6 (Single meCpG). One-way ANOVA followed by Bonferroni post-hoc pairwise comparisons. All groups with gRNA are significantly different than those without gRNA. ****P < 0.0001, ***P < 0.001. (**C**) MSRE-qPCR timecourse of HEK293T cells transfected with Cas9+gRNA and no repair template. *n* = 3 experiments. (**D**) MSRE-qPCR timecourse of HEK293T cells transfected with Cas9 and ssoligo repair templates. *n* = 3 experiments. Two-way ANOVA shows significant interaction between time post transfection and template (*P* = 0.0010). Comparisons are made to Cas9 without gRNA transfected group. ****P < 0.0001, **P < 0.01, *P < 0.05.

We first tested the hypothesis that DNA methylation can be copied from an exogenous repair template by designing 190 bp single stranded DNA (ssDNA) repair templates that were homologous to a region within the *C9orf72* CpG island (Figure 1A). These templates were either unmethylated at all 10 CpG sites, methylated at all 10 CpG sites or only methylated at 1 CpG site (Figure 1A). We also included a non-homologous template (NH) to test whether DNA double strand breaks in the absence of HDR, which are presumably repaired via NHEJ, could also lead to DNA methylation. HEK293T cells were transfected with a plasmid encoding for SpCas9 nuclease with or without a gRNA specific to the *C9orf72* promoter, together with the indicated repair templates. Methylation was measured at a CpG site in the promoter region (Figure 1A) using a previously developed methylation sensitive restriction enzyme digest qPCR assay (MSRE-qPCR) (4). This assay provides a quantitative estimate of methylation at the *C9orf72* promoter based on DNA resistance to digestion by the methylation-sensitive restriction enzyme HhaI. The primers used anneal outside the repair templates and thus can only amplify genomic DNA. In the absence of gRNA, there were no measurable changes in HhaI resistance among the groups, indicating little methylation is normally present at this site (Figure 1B). Cells transfected with plasmids encoding Cas9, a gRNA targeting *C9orf72* and a non-homologous repair template exhibited an increase in HhaI resistance (Figure 1B), indicating that dsDNA breaks followed by NHEJ may result in DNA methylation. To measure NHEJ, a T7 endonuclease assay was used that digests mismatched PCR amplicons (40), and PCR followed by sanger sequencing was used to characterize the specific indel patterns in each group. Indel formation was highest in the NH group (Supplemental Figure S1), raising the possibility that some of the HhaI resistance in this group may be due to rare deletion of the HhaI site or generation of damaged DNA (e.g. nicked or single stranded) that is resistant to HhaI digestion.

When comparing the groups transfected with homologous templates, those receiving methylated template had higher levels of HhaI resistance than cells transfected with unmethylated template (Figure 1B). Furthermore, the group receiving the single methylated CpG template, corresponding to the HhaI restriction enzyme site used in the MSRE-qPCR assay, exhibited methylation with similar efficiency as the fully methylated template (Figure 1B). These results suggest that methylated exogenous repair templates can promote DNA methylation of the endogenous *C9orf72* promoter, and a single methylated CpG is sufficient to induce methylation of this site. A 2 bp mutation was included in the repair templates to create an SspI restriction enzyme site that is not normally found in this region. This mutation blocks the CRISPR PAM site to disallow repeat cutting of edited loci and enables monitoring of HDR efficiency by PCR and restriction enzyme digest. HDR rates were comparable between unmethylated and methylated template groups (Supplemental Figure S1), suggesting that differences in repair efficiency do not account for the observed differences in HhaI resistance.

Next, we measured HhaI resistance over time in culture to see if methylation induced via double strand break repair pathways was stable. HhaI resistance induced by double strand break alone (Cas9+gRNA and no repair template) was transient, with levels decreasing from ∼7% to 1.5% over the course of 5 days in culture (Figure 1C). Similarly, HhaI resistance in the methylated oligo repair template group was also transient, although it took 8 days post transfection for levels to return to baseline (Figure 1D). These results suggest that while methylation can be induced via HDR with short single stranded methylated repair templates, it is not maintained during multiple rounds of DNA replication.

### Longer double stranded templates improve methylation efficiency and stability

**Figure 2.**
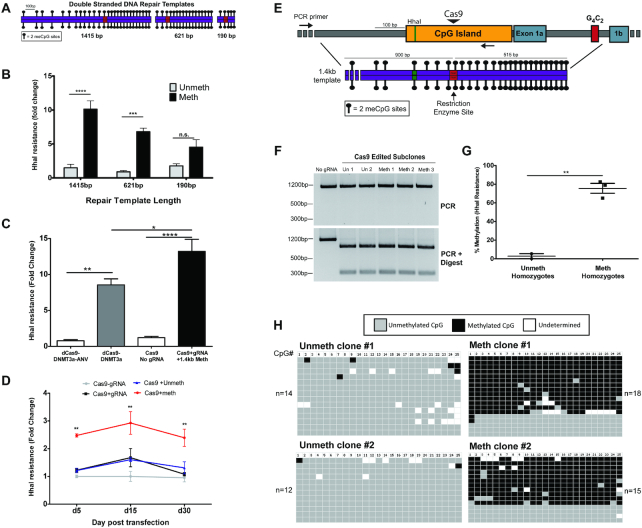
Editing DNA methylation with double-stranded repair templates. (**A**) Diagram of double stranded DNA repair templates (either fully methylated or fully unmethylated at CpG sites) homologous to the *C9orf72* promoter. Red box indicates a 2 bp substitution that generates a restriction enzyme site and mutates the PAM sequence. Figure drawn to scale. (**B**) MSRE-qPCR assay to measure DNA methylation using a long amplicon that anneals outside the repair templates. HEK293T cells were transfected with CRISPR/Cas9+gRNA and the indicated templates. Cells were puromycin selected for 2 days and collected at day 4 post transfection. *n* = 3 experiments; two-way ANOVA shows significant interaction between template length and template methylation status (*P* = 0.0071). Bonfferoni post-hoc test between unmeth and meth template groups ****P < 0.0001, *** *P* < 0.001. (**C**) Comparison of methylation efficiency between HARDEN method and dCas9-DNMT3a fusion protein construct. HEK293T cells were transfected with indicated plasmids and methylation was measured 2 days post transfection using long amplicon MSRE-qPCR. n = 3 experiments. One-way ANOVA (*P* < 0.0001) followed by Bonferroni post-hoc comparison of indicated groups. ****P < 0.0001, **P < 0.01, *P < 0.05. (**D**) Timecourse of methylation measured via long amplicon MSRE-qPCR using 1.4kb dsDNA repair templates. n = 3 experiments. Two-way ANOVA (Group *P* < 0.0001; day post transfection *P* = 0.0343) followed by Bonferroni post-hoc comparison between unmethylated template and methylated template groups. **P < 0.01. (**E**) Diagram of *C9orf72* promoter region indicating the position of the repair template used to generate stably methylated HEK293T clones. Red box indicates a 2 bp substitution that generates a restriction enzyme site and mutates the PAM sequence. Figure drawn to scale. (**F**) PCR and digest of HEK293T clonal lines that were repaired using the 1.4kb dsDNA repair templates. Cells were collected ∼5 weeks post transfection. (**G**) MSRE-qPCR of clonal cell lines that were homozygous for editing with either the unmethylated (*n* = 2) or methylated 1.4kb repair template (*n* = 3). Cells were collected ∼5 weeks post transfection. Two-tailed *t*-test; *P* = 0.0020. (**H**) Bisulfite amplicon sequencing of homozygous clonal cell lines edited with the unmethylated or methylated 1.4kb repair templates. Cells were collected ∼5 weeks post transfection. Each row is an individual DNA molecule and columns are individual CpG dinucleotides. Grey boxes indicate unmethylated CpGs, black boxes indicate methylated CpGs and white boxes are undetermined due to poor conversion or poor sequencing quality.

While it appeared that methylation of the endogenous *C9orf72* promoter could be induced upon HDR with a methylated exogenous oligonucleotide repair template, methylation appeared to be relatively inefficient and transient. To enhance DNA methylation efficiency and stability, we tested the ability to introduce DNA methylation via HDR using longer double stranded DNA (dsDNA) repair templates. *In vitro* methylated dsDNA repair templates with 190 bp (10 CpGs), 621 bp (35 CpGs) and 1.4 kb (53 CpGs) total homology to the *C9orf72* promoter were generated (Supplemental Table S2, Figure 2A). Unmethylated versus fully methylated repair templates were co-transfected with plasmids encoding CRISPR-Cas9 components into HEK239T cells. After 4 days, methylation was tested using a long amplicon MSRE-qPCR assay that only amplifies genomic DNA and not the repair templates. Longer dsDNA templates induced more HhaI resistance in a length dependent manner, with the longest repair template group showing more than two fold higher methylation levels than the shortest template group (Figure 2B). Bisulfite amplicon sequencing of HhaI resistant DNA was then carried out to characterize DNA methylation patterns in edited cells. This analysis revealed that HhaI resistant DNA was highly enriched for HDR sequences that were densely methylated (Supplemental Figure S2). Sequences containing indels, and thus likely repaired via NHEJ, were also enriched for methylation but at much lower levels than DNA repaired via HDR (Supplemental Figure S2). Additionally, HDR efficiency of the different template lengths does not explain the observed differences in DNA methylation, as the shortest 190bp methylated repair template resulted in the most HDR, but the least amount of DNA methylation (Supplemental Figure S3). We also compared methylation induced by the methylated 190 bp dsDNA repair template versus the single stranded 190 bp oligonucleotide repair template and found a minor but non-significant increase in HhaI resistance associated with the dsDNA template (Supplemental Figure S4). This suggests that DNA strandedness does not completely account for the increase in methylation efficiency when using longer dsDNA repair templates. Together, these results suggest that the length of the dsDNA repair template was the main factor that allowed for more efficient induction of DNA methylation.

Next, the HARDEN method of targeted DNA methylation was compared to the previously developed dead Cas9 (dCas9) DNMT3a catalytic domain fusion protein construct (15). HEK293T cells were transfected with either Cas9+gRNA to *C9orf72* and the 1.4kb dsDNA methylated repair template or the dCas9-DNMT3a construct with the same gRNA. As negative controls, cells were transfected with catalytically inactive DNMT-dCas9 fusion enzyme (dCas9-DNMT3a-ANV) or Cas9 plasmid without gRNA. In control groups, there was very little detectable methylation using MSRE-qPCR (Figure 2C). Robust levels of methylation were induced using either dCas9-DNMT3a or HARDEN; however, the HARDEN method induced ∼50% more methylation and thus has slightly improved efficiency at this locus. These results indicate that HARDEN may be an effective alternative method for targeted DNA methylation of the *C9orf72* locus.

We also tested whether methylation copied from a long dsDNA repair template is stably maintained after cell division, as this is important for targeted methylation editing applications. Cells were transfected with Cas9 and gRNA targeting *C9orf72* and either non-homologous repair template, unmethylated 1.4 kb dsDNA repair template or methylated 1.4 kb dsDNA repair template. As a control, cells transfected with Cas9 plasmid without gRNA and with a non-homologous repair template were used. Methylation was measured by MSRE-qPCR at the indicated days post transfection. Cells that were transfected with methylated template maintained higher methylation levels than controls and remained stable throughout the 30 day time course (Figure 2D). Additionally, cells transfected with either the unmethylated or methylated 1.4 kb repair templates were sub-cloned and screened for clonal lines that underwent HDR using the repair template via PCR and restriction enzyme digest (Figure 2E, F). Clones were passaged from single cells and analyzed ∼5 weeks after transfection of CRISPR components and repair template. 9 out of 13 clones (69.2%) that were repaired with the methylated template acquired >10% methylation, compared to only 1 clone out of 16 (6.3%) repaired with the unmethylated template (Supplemental Figure S5) as measured by MSRE-qPCR. Furthermore, all three clones that were repaired with the methylated template on both alleles became hypermethylated, with levels of methylation ranging from 60 to 80% by both MSRE-qPCR and bisulfite sequencing of cloned amplicons (Figure 2G, H). Thus, repair with methylated dsDNA repair templates promotes stable and dense methylation of the *C9orf72* promoter.

### DNA methylation is acquired via homology directed repair

**Figure 3.**
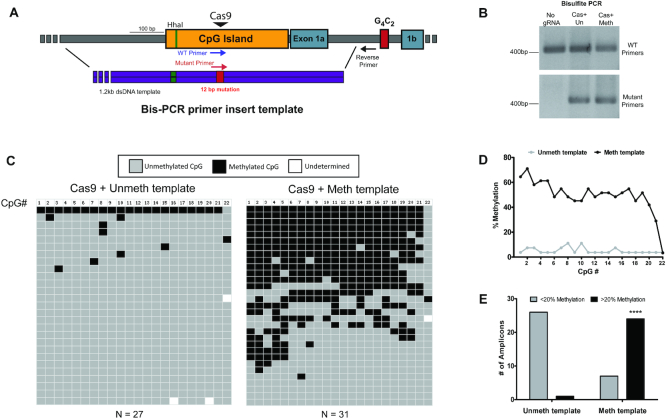
DNA methylation is associated with Homology Directed Repair (**A**) Diagram showing the targeted region of *C9orf72*. Purple arrows indicate primers that amplify wild type DNA, whereas the red arrow indicates the primer that specifically amplifies a 12bp mutation that was included in the repair template. Figure drawn to scale. (**B**) Bisulfite PCR using the primers depicted in (A). Representative gel from two biological replicates. (**C**) Bisulfite amplicon sequencing from two biological replicates (Unmeth: *n* = 27 amplicons, Meth: *n* = 31 amplicons). Each row is an individual cloned and sequenced DNA molecule and columns are individual CpG dinucleotides. Grey boxes indicate unmethylated CpGs, black boxes indicate methylated CpGs and white boxes are undetermined due to poor conversion or poor sequencing quality. (**D**) Quantification of the percentage of methylated CpGs in (C). *n* = 2 biological replicates. Two-tailed *t*-test; *P* = 0.0109. (**E**) Quantification of the number of amplicons with less than or >20% methylation. Data are from two biological replicates. Fisher's exact test, *P* < 0.0001.

Next, the underlying mechanisms of HARDEN were explored to better understand the efficiency and required proteins for this process. The first question tested was what percentage of DNA molecules that are repaired with an exogenous methylated repair template actually undergo copying of methylation into the endogenous DNA. To address this question, a ∼1200 bp dsDNA repair template was designed to insert a 12 bp mutation that would serve as a primer site to specifically amplify genomic DNA in which the mutation is incorporated via HDR (Figure 3A). HEK293T cells were transfected with CRISPR components and either an unmethylated or methylated version of this template. Bisulfite PCR of genomic DNA from transfected cells showed that the mutation specific PCR only amplified DNA from cells that were co-transfected with repair template (Figure 3B). Bisulfite sequencing of these cloned amplicons revealed that DNA repaired with the unmethylated template was rarely methylated, with low levels of methylation across the amplified region (Figure 3C, D) and only 1/27 (3.7%) amplicons having at least 20% methylation (Figure 3E). In comparison, DNA repaired with the methylated template was methylated >40% at almost all CpGs (Figure 3C, D) and had 24/31 (77.4%) highly methylated amplicons (Figure 3E). Moreover, bisulfite sequencing revealed that DNA methylation in the setting of HDR did not induce DNA methylation of the last 3′ CpG site within the bisulfite amplicon (CpG site #22, Figure 3C, D). This is notable because the repair template covered the entire bisulfite amplicon except this 3′ CpG site, consistent with our hypothesis that DNA methylation is copied in a templated manner. Thus, repair with a methylated homologous template facilitates methylation of the endogenous locus.

Next, siRNA knockdowns of DNA methyltransferase enzymes were carried out to determine which DNMTs are required for methylation during DNA repair. siRNA against either DNMT1, DNMT3a, DNMT3b and the DNMT1 recruiting factor UHRF1 were first transfected into HEK293T cells, and 24 h later, the CRISPR components with either no template or with the methylated 1.4kb repair template were introduced. Methylation was measured by MSRE-qPCR following an additional 48 h incubation period. In the context of NHEJ (no repair template), knockdown of only DNMT3b significantly reduced methylation (Supplemental Figure S6A). In contrast, knockdown of any DNMT or UHRF1 resulted in ∼40–50% decrease in the amount of methylation acquired using HARDEN (Supplemental Figure S6B). These results suggest that DNMT3b may be specifically required for NHEJ induced DNA methylation, whereas there may be non-redundant roles for DNMTs and UHRF1 for the establishment and/or maintenance of methylation during HDR.

### Genome wide methylation of epigenetically edited cells

**Figure 4.**
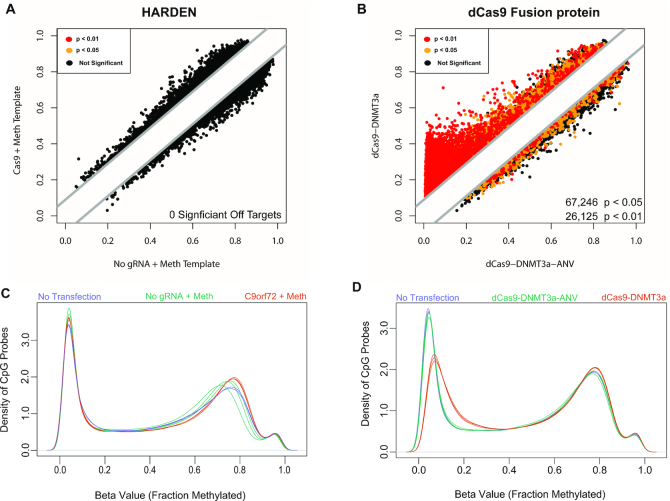
Genome-wide methylation analysis of HARDEN and Cas9-DNMT3a based methods. (**A**) Methylation values from HEK293T cells transfected with Cas9+gRNA to *C9orf72* and a methylated repair template vs those without gRNA. Methylation was measured on an Illumina Epic Methylation Array with ∼800,000 probes. Cells were puromycin selected and collected at day 4 post transfection. *n* = 3 biological replicates; Colored dots indicate differentially methylated probes calculated using the Benjamini–Hochberg procedure. Black = not significant (adjusted *P* > 0.05); yellow = adjusted *P* < 0.05; red = adjusted *P* < 0.01. (**B**) Methylation values from HEK293T cells transfected with either dCas9-DNMT3a or catalytically dead dCas9-DNMT3-ANV along with gRNA to *C9orf72*. Cells were puromycin selected and collected at day 4 post transfection. *n* = 3 biological replicates. Methylation was measured on an Illumina Epic Methylation Array with ∼800,000 probes. Colored dots indicate differentially methylated probes calculated using the Benjamini–Hochberg procedure. Black = not significant (adjusted *P* > 0.05); yellow = adjusted *P* < 0.05; red = adjusted *P* < 0.01. (**C**) Raw methylation values versus the density of probes genome-wide for non-transfected cells (*n* = 2), Cas9+gRNA and methylated template (*n* = 3) or Cas9 without gRNA and methylated template (*n* = 3). (**D**) Raw methylation values vs the density of probes genome-wide for non-transfected cells (*n* = 2), dCas9-DNMT3a+gRNA to C9orf72 (*n* = 3) or catalytically dead dCas9-DNMT3-ANV+gRNA to *C9orf72* (*n* = 3).

The previous results demonstrate that HARDEN can be a useful method for targeted methylation of *C9orf72*. Next, the potential genome-wide off target effects of HARDEN were explored and compared to the dCas9-DNMT3a catalytic domain fusion protein construct. Because HARDEN utilizes both the 20 bp gRNA sequence and a homologous repair template to induce methylation, we hypothesized that HARDEN would be highly specific for the targeted locus. To test this, HEK293T cells were transfected with either the dCas9-DNMT3a construct, or CRISPR components and methylated repair template (C9orf72+Meth). As negative controls, catalytically inactive dCas9 enzyme (dCas9-DNMT3a-ANV) or Cas9 plasmid without gRNA but with a methylated repair template (No gRNA+Meth) were used. Non-transfected cells were also included as an additional control to ensure transfection did not perturb global DNA methylation levels. Genome-wide methylation analysis was measured using genome-wide arrays that detect DNA methylation at ∼800,000 CpG sites. No statistically significant off target effects were detected using the HARDEN method (Figure 4A). This is in contrast to the dCas9-DNMT3a fusion construct which resulted in massive off target methylation (67,246 sites with *P* < 0.05) when compared to the dCas9-DNMT3a-ANV construct (Figure 4B). When looking at the raw methylation values across the genome, there was no difference in methylation at lowly methylated sites between non-transfected cells or cells transfected with HARDEN components (Figure 4C). However, cells transfected with dCas9-DNMT3a showed a pronounced gain in methylation at lowly methylated sites compared to non-transfected cells and cells transfected with dCas9-DNMT3a-ANV (Figure 4D). These results suggest that HARDEN is a highly specific method for DNA methylation editing and confirms previous findings that dCas9-DNMT3a fusion proteins have massive global off-target effects.

### Editing of methylation in ALS patient derived iPSCs

**Figure 5.**
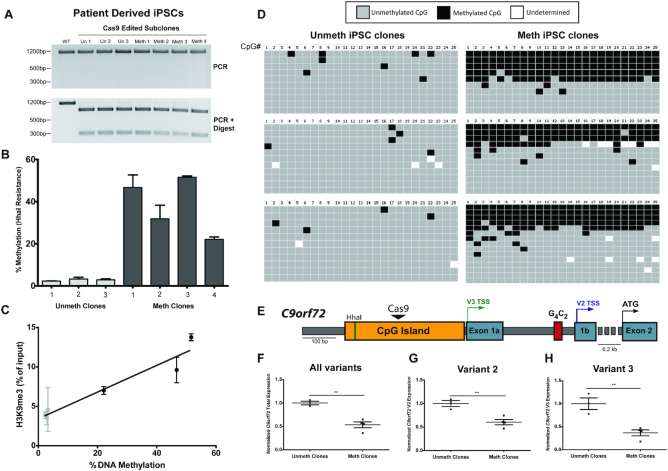
Editing of DNA methylation in ALS patient derived iPSCs. (**A**) PCR and digest of clonal ALS patient derived iPSCs that were repaired using 1.4 kb double stranded dsDNA repair templates. (**B**) MSRE-qPCR to measure DNA methylation of clonal iPSCs that were homozygous for editing with the repair template. Cells were puromycin selected for 2 days, subcloned at day 7 and collected at ∼6 weeks post transfection. *n* = 2 measurements per cell line and 3–4 cell lines per group. (**C**) Correlation between H3K9 trimethylation levels measured by ChIP-qPCR (*n* = 2 experiments and three cell lines per group) and DNA methylation measured by MSRE-qPCR. Linear regression, *R*^2^ = 0.9242; *P* = 0.0022. (**D**) Bisulfite amplicon sequencing from unmethylated or methylated clonal iPSCs (*n* = 3 cell lines per group). Each row is an individual cloned and sequenced DNA molecule and columns are individual CpG dinucleotides. Grey boxes indicate unmethylated CpGs, black boxes indicate methylated CpGs and white boxes are undetermined due to poor conversion or poor sequencing quality. (**E**) Diagram of *C9orf72* gene structure. Black triangle indicates the Cas9 cut site, colored arrows indicate transcription start site and black arrow indicates the protein coding start site. Figure drawn to scale. (**F**) RT-qPCR of all *C9orf72* variants in methylated or unmethylated clonal iPSCs. Unmeth cell lines: *n* = 3; Meth cell lines: *n* = 4. Two-tailed *t*-test, *P* = 0.0021 (**G**) RT-qPCR of *C9orf72* variant 2 in methylated or unmethylated clonal iPSCs. Unmeth cell lines: *n* = 3; Meth cell lines: n = 4. Two-tailed *t*-test, *P* = 0.0056. (**H**) RT-qPCR of *C9orf72* variant 3 in methylated or unmethylated clonal iPSCs. Unmeth cell lines: *n* = 3; Meth cell lines: *n* = 4. Two-tailed t-test, *P* = 0.0048.

ALS and FTD patients with the *C9orf72* repeat expansion can exhibit promoter hypermethylation, which is thought to contribute to transcriptional repression of the *C9orf72* gene (4). To model this aspect of ALS/FTD, HARDEN was applied to previously characterized patient derived iPSCs (7) harboring the repeat expansion but with limited *C9orf72* promoter methylation (Supplemental Figure S7). IPSCs were transfected with CRISPR components and either unmethylated and or methylated 1.4 kb dsDNA templates. Clonal lines were isolated and tested for incorporation of the PAM mutation via HDR using restriction enzyme digestion of PCR amplicons (Figure 5A), and methylation of edited clones was measured using both MSRE-qPCR and bisulfite amplicon sequencing. Targeted methylation of iPSCs was somewhat less efficient than in HEK293Ts, but stably methylated clones were obtained with methylation levels of ∼20–50% (Figure 5B–D). Additionally, chromatin immunoprecipitation (ChIP) was performed on clonal iPSCs using an antibody against the repressive histone mark H3K9 trimethylation (Figure 5C). iPSCs that acquired DNA methylation via HARDEN also gained H3K9 trimethylation, indicating a switch to a repressive chromatin state, and this increase was specific for the *C9orf72* locus (Figure 5C, Supplemental Figure S8).

Next, RNA was extracted from these edited iPSCs and mRNA levels of the major *C9orf72* isoforms were measured via RT-qPCR (Figure 5E). Methylated iPSC clones had reduced mRNA levels by ∼50%, with the largest effect seen for variant 3 (Figure 5H), which has a transcription start site closest to the targeted CpG island. These results indicate that hypermethylation of the *C9orf72* promoter CpG island does indeed cause a reduction of *C9orf72* expression, confirming previous reports that were based only on correlative analysis (4,5). Thus, HARDEN can be used to generate a disease model of aberrant methylation and allows for defining the direct effect of DNA methylation on gene expression at this locus.

### Targeted DNA methylation of amyloid precursor protein (*APP*)

**Figure 6.**
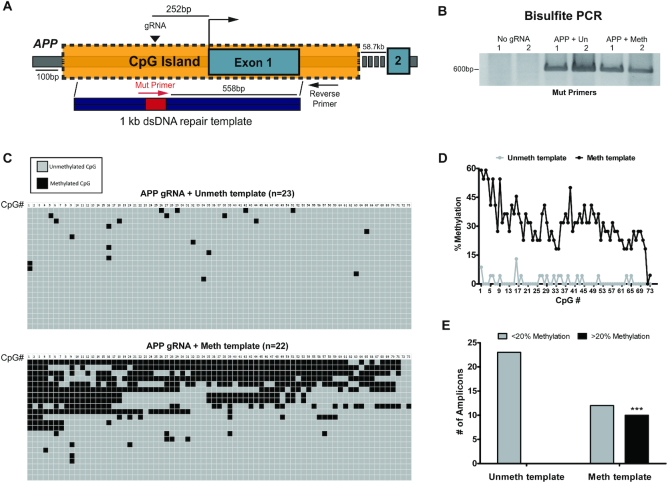
Targeted DNA methylation of APP. (**A**) Diagram of APP gene locus showing CpG island, gRNA targeting site (black triangle) and bisulfite primers (Red and black arrows) used to amplify only DNA that incorporates a 12bp mutation (red box) from the template. Figure is drawn to scale, except for primers which are for illustrative purposes. (**B**) Bisulfite PCR of HEK293T cells transfected with No gRNA plasmid, APP gRNA + unmethylated template or APP gRNA + methylated template. *n* = 2 biological replicates. Primer location is indicated in (A). (**C**) Bisulfite sequencing of cloned amplicons from (B). Each column represents a CpG site in the ∼600 bp amplicon. Grey boxes indicate unmethylated cytosines whereas black boxes represent methylated cytosines. *n* = 23 sequences for APP gRNA+ unmethylated template group and *n* = 22 sequences for APP gRNA+ methylated template group. (**D**) Quantification of mean methylation at each CpG site in the amplicon (73 total CpG sites) depicted in (C). *n* = 23 sequences from unmethylated template group and *n* = 22 sequences from methylated template group. (**E**) Number of highly methylated amplicons (>20% methylation) from bisulfite sequencing depicted in (C). *n* = 23 sequences for unmethylated template group and *n* = 22 for methylated template group. Ten amplicons are highly methylated in the methylated template group versus 0 amplicons in the unmethylated template group. ***P = 0.0002; Fisher's exact test (two-sided).

To determine if HARDEN can be applied to another locus besides *C9orf72*, we targeted a large CpG island in the promoter region of *APP*. *APP* encodes amyloid precursor protein, a key protein involved in Alzheimer's disease (AD) pathogenesis (42). As a proof of principle, we applied the HARDEN system to *APP* by inducing a double strand break with Cas9 and providing a methylated repair template (Figure 6A). The repair template also included a 12 base pair mutation that allows for bisulfite PCR amplification of only the DNA that has undergone HDR using the repair template (Figure 6B). Cloning of bisulfite amplicons repaired with an unmethylated or methylated template was then carried out to assess the DNA methylation pattern of repaired DNA (Figure 6C). This analysis revealed that DNA repaired with the methylated template is highly enriched for methylation, with the CpG sites closest to the cut site exhibiting levels of methylation exceeding 50% (Figure 6C-D). In some clones, methylation was copied >500 bp downstream from the cut site (Figure 6C, D). In sum, ∼45% of amplicons (10/22) gain dense DNA methylation when repaired with a methylated template, compared to no amplicons (0/23) repaired with the unmethylated template (Figure 6E). These results are consistent with the above experiments at *C9orf72* and again suggests that DNA methylation can be copied during HDR. Furthermore, these data confirm that HARDEN is a generalizable method that can be applied to other genetic loci besides *C9orf72*.

## DISCUSSION

This study highlights a novel method for targeted DNA methylation and applies it to generate cellular diseases models of genes involved in neurodegeneration. In doing so, several new findings about the interplay between DNA damage repair pathways and DNA methylation were explored. First, the results presented here suggest that a targeted double strand break can induce DNA methylation of an endogenous CpG island, albeit at low frequency. Previous studies have demonstrated that repair of dsDNA breaks in a GFP-based reporter construct can lead to DNA methylation following repair by either NHEJ (43) or HDR (33,36–38). This study demonstrates that this process also occurs at an endogenous gene, although methylation acquired following NHEJ is sparse and not maintained through multiple rounds of cell division. We note the HhaI resistance assay may be over-estimating DNA methylation induced by NHEJ, as HhaI-based methylation sensitive restriction enzyme assays can potentially be partially blocked by factors other than DNA methylation such as regions of single stranded DNA (44,45). However, bisulfite sequencing revealed that a subset of sequences with indels do appear to acquire limited DNA methylation. These results indicate that localized DNA methylation may serve as a transient marker of dsDNA breaks, perhaps to prevent transcription and promote stability of potentially mutagenized DNA.

Perhaps the most important finding presented here is that methylation appears to be copied from either a single or double stranded DNA template through the process of HDR, including from oligonucleotides harboring a single methylated CpG site. This level of specificity could be useful to study the effect of a particular CpG site on transcription factor binding. Longer double stranded templates seem to induce more methylation than short single stranded oligonucleotide templates and methylation induced via dsDNA templates can be stably maintained over numerous cell divisions in both HEK293T cells and iPSCs. Our results suggest that template length is more important than strandedness for inducing methylation, as there is only a small, non-significant increase in efficiency between 190 bp single stranded templates and the corresponding 190 bp dsDNA template. In contrast, there are more sizeable observed differences in efficiency between 190 bp, ∼600 bp and ∼1.4 kb dsDNA templates. It is not clear whether this effect is due to the overall increase in the number of methylated CpGs or if the longer templates include key CpG sites necessary for stable methylation of the *C9orf72* promoter. There is evidence, for example, that specific transcription factor binding sites may be more important than the total number of methylated CpGs for determining promoter methylation state (46).

**Figure 7.**
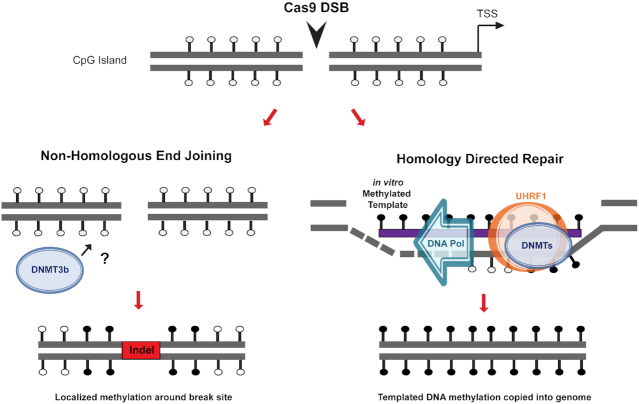
Proposed mechanism of targeted methylation via endogenous DNA repair. Double strand break within a CpG island leads to repair by either Non-Homologous End Joining (NHEJ) or Homology Directed Repair (HDR). Without repair template present, the cell undergoes NHEJ and in rare cases can utilize DNA methyltransferase 3b (DNMT3b) by an unknown mechanism to deposit methylation around the break site. NHEJ is error prone, leading to insertions or deletions (indels), and methylation is not stably maintained. If the cell is provided with an exogenous *in vitro* methylated repair template, however, the break can be repaired with HDR. During this process, recruitment of UHRF1 and DNMTs leads to copying of methylation from the repair template into the endogenous DNA.

Knockdown of DNA methyltransferase enzymes revealed that both *de novo* (DNMT3a and 3b) and maintenance methyltransferases (DNMT1) as well as UHRF1 are required for efficient methylation using HARDEN, whereas DNMT3b seems to be the most prominent factor involved in methylation associated with NHEJ. These enzymes have previously been shown to assemble at double strand DNA break sites (32,33,36), and UHRF1 is thought to be important for double strand break repair in addition to its role in maintenance methylation (47). Thus, it appears that an interplay of these enzymes and cofactors function in concert to promote methylation during DNA damage repair, although they may function in different repair sub-pathways. We propose a model in which double strand break followed by repair via NHEJ can lead to recruitment of DNMT3b and subsequent methylation of nearby CpG sites (Figure 7, left). Methylation following NHEJ is transient and is not faithfully copied during DNA replication. On the other hand, an exogenous methylated template can be used during HDR for copying of methylation into the endogenous DNA (Figure 7, right). Methylation induced via HDR requires both *de novo* DNMTs and the methylation maintenance complex (DNMT1/UHFR1) for induction and replication of methylation over numerous rounds of cell division.

HARDEN was also compared to the dCas9-DNMT3a fusion protein, and it appears that HARDEN has slightly better on-target efficiency at *C9orf72* than the dCas9-DNMT3a construct. Importantly, HARDEN has no detectable off-target effects, whereas the dCas9-DNMT3a fusion protein induces tens of thousands of off-target methylation changes. While only the off-target effects of a single gRNA are compared here, other studies have documented that dCas9-DNMT3a fusion proteins have large off-target effects irrespective of the gRNA used (18,20).

HARDEN can also be used to make stably methylated cell lines that model epigenetic alterations seen in ALS/FTD patients with the *C9orf72* repeat expansion (4,5,48). Indeed, this study confirms for the first time that the endogenous *C9orf72* promoter CpG island directly regulates expression of this gene and that DNA methylation of this region leads to an increase in histone H3K9 trimethylation. Interestingly, the overall levels of methylation in clonal iPSCs are lower than in clonal HEK293Ts. This could be due to differences in active demethylation between the cell types or selective pressure against fully methylated *C9orf72* in iPSCs. It is known that iPSCs require active demethylation via TET enzymes to maintain pluripotency (49).

We also demonstrate that HARDEN is not specific to the *C9orf72* locus, but can be used to methylate the neurodegenerative disease gene *APP* as well. APP is cleaved by proteases to generate amyloid beta peptides, which are a major component of amyloid plaques found in AD patient post mortem brain tissue (42). Amyloid beta production is thought to play a central role in AD pathogenesis, as mutations in *APP* that increase amyloid beta levels are known to cause dominantly inherited early-onset AD (50). Several studies have investigated the DNA methylation status of the CpG island upstream of *APP* but have come to differing conclusions as to whether *APP* is differentially methylated in AD patients (51–54). Thus, the application of HARDEN at this locus could be useful for generating cellular models of DNA methylation to explore the effects of methylation on *APP* expression.

We acknowledge that HARDEN may not the best targeted DNA methylation technique for some applications. Since HARDEN appears to be linked to HDR, methylation efficiency is dependent on HDR rates, which can be low or absent in certain contexts. Furthermore, since stable methylation editing requires both a gRNA and repair template, multiplexing to target numerous genes at once would be labor intensive and multiple double strand breaks could be cytotoxic. A final limitation is that in order to prevent cutting of the repair template, a PAM mutation is required and thus scarless methylation editing without changing the DNA sequence is difficult. Nevertheless, the cellular models established here using HARDEN can be utilized to better understand the molecular consequences of DNA methylation in the context of neurodegenerative disease.

## DATA AVAILABILITY

Genome-wide DNA methylation array data is publically available at GEO (GSE134996).

## Supplementary Material

gkz979_Supplemental_File
