# Supplementary material for: Targeted DNA methylation of neurodegenerative disease genes via homology directed repair
Source: Nucleic Acids Res. 2019 Nov 4;47(22):11609–22. doi: 10.1093/nar/gkz979 (PMC7145628; doi:10.1093/nar/gkz979)
Supplement: gkz979_Supplemental_File [file gkz979_supplemental_file.pdf]

Supplemental Information for *Targeted DNA methylation of neurodegenerative disease genes via homology directed repair*

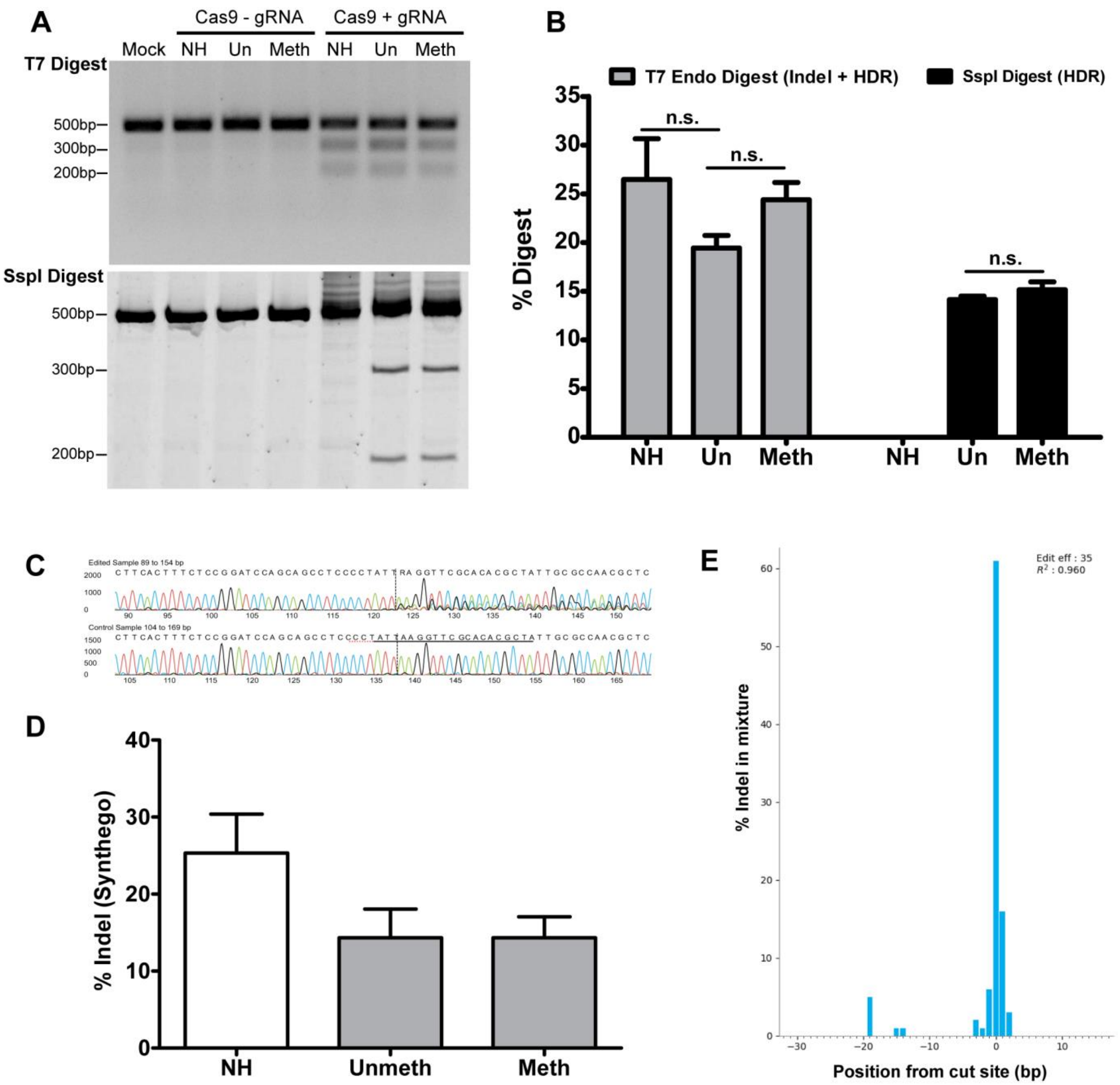

Supplemental Figure 1: Indel and HDR rates of HEK293T cells edited with ssOligo templates. **A)** Representative PCR + Digest with T7 endonuclease (top gel) or SspI (bottom). Digest with T7 estimates the total amount of mismatched DNA (Indels and copying of the 2bp PAM mutation via HDR) whereas SspI digest estimates the rate of HDR (copying of the 2bp PAM mutation creates SspI restriction enzyme site). n= 3 experiments. **B)** Quantification of T7 and SspI Digest assays. n = 3 experiments. Two-way ANOVA shows significant interaction between

sample group and digest assay (p = 0.0004). Bonferroni post-hoc tests were carried out between groups within each assay. **C)** Example Sanger sequencing trace from Cas9+gRNA+NH group (top) compared to Cas9-gRNA+NH group (bottom). Black dotted line indicates CRISPR/cas9 cut site and black underline indicates gRNA site. Analysis was carried out using Synthego ICE software on PCR amplicons from HEK293T cells shown in Figure 1B. **D)** Percent insertions or deletions (indel) induced by CRISPR/Cas9 cutting as measured using Synthego ICE software on PCR amplicons from HEK293T cells shown in Figure 1B. n = 3 experiments per group. **E)** Example quantification of indel sizes in the Cas9+gRNA+NH group. The maximum deletion size was 19bp and maximum insertion size detected was 2bp. No insertion of the non-homologous template was detected. Analysis was carried out using Synthego software on PCR amplicons from HEK293T cells shown in Figure 1B. n = 3 experiments.

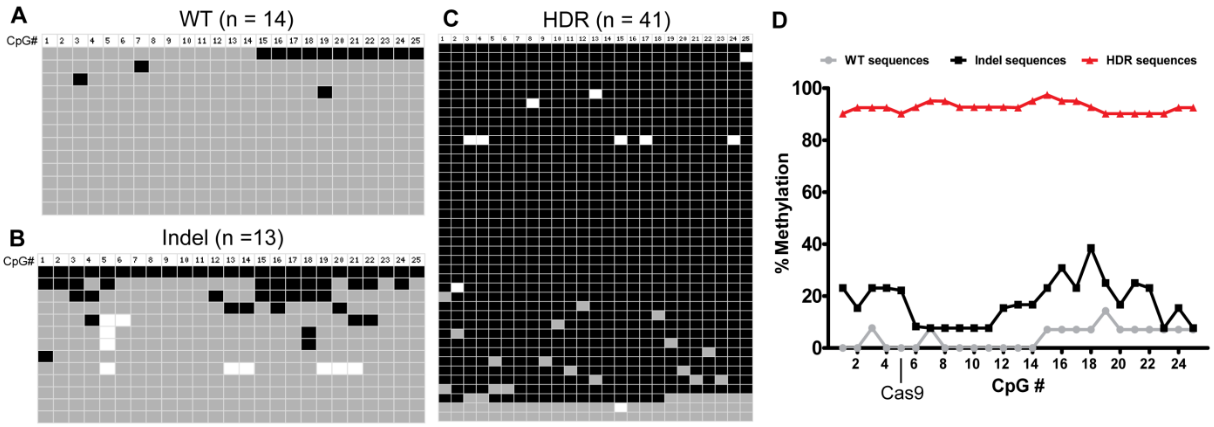

Supplemental Figure 2: Bisulfite amplicon sequencing of HhaI resistant DNA. DNA from HEK293T cells transfected with Cas9+gRNA+621bp methylated template (Figure 2B) was digested with HhaI and bisulfite amplicon cloning was carried out on HhaI resistant DNA. Sequencing results were analyzed with BISMA software and each sequence was classified as to whether it matched the WT reference sequence **(A)**, contained indels **(B)** or contained the 2bp PAM mutation that indicates HDR with the methylated repair template occurred **(C).** Grey boxes indicate unmethylated CpG sites; black boxes indicate methylated CpG sites; white boxes indicate the CpG site was mutated or had poor sequencing quality. **D)** Quantification of bisulfite amplicon sequencing shown in A-C. Cas9 cut site is nearest to CpG #5, as indicated.

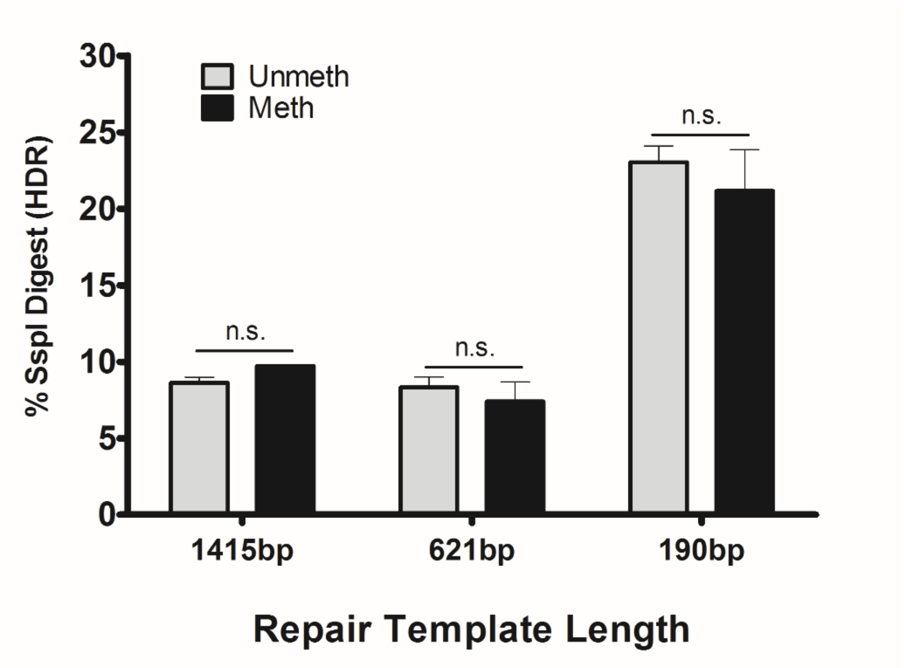

Supplemental Figure 3: Comparison of HDR efficiency in different length templates. Quantification of PCR + SspI Digest in HEK293T cells transfected with the indicated templates. Cells were puromycin selected for 2 days and collected at day 4 post transfection. n = 3 experiments. Two-way ANOVA shows template size has significant effect ($p < 0.0001$) whereas methylation status does not ($p = 0.6110$). Bonferroni post-hoc tests were carried out between unmeth and meth groups at each template length.

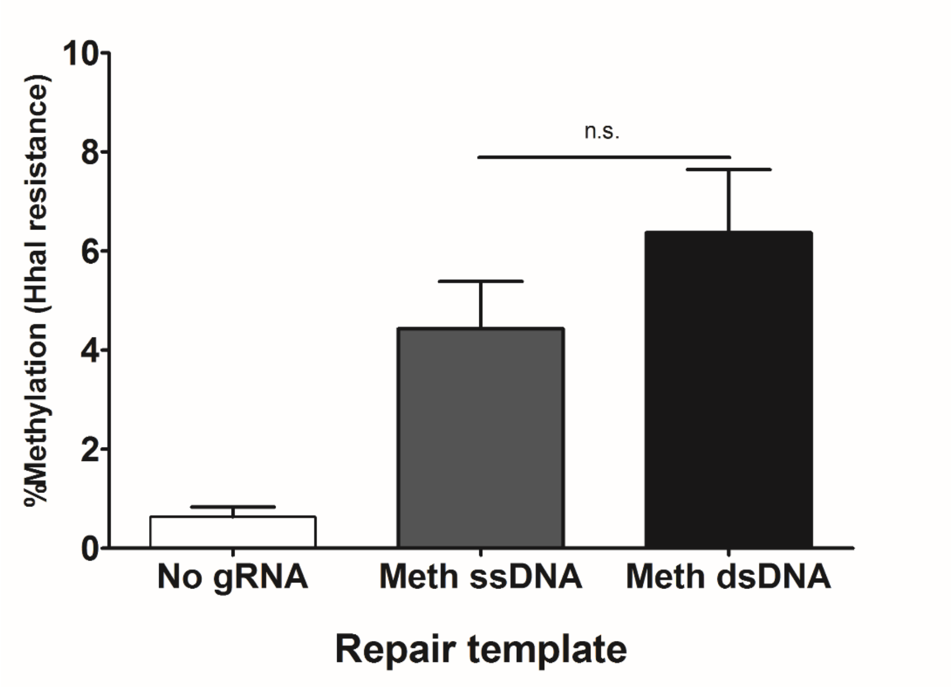

Supplemental Figure 4: Comparison of ssDNA vs dsDNA templates. MSRE-qPCR of HEK293T cells transfected with either 190bp ssOligo or 190bp dsDNA methylated templates and the CRISPR/cas9 components. Cells were puromycin selected for 2 days and collected at day 4 post transfection. n = 3 experiments. One-way ANOVA ($p = 0.0126$) followed by Bonferroni post-hoc test.

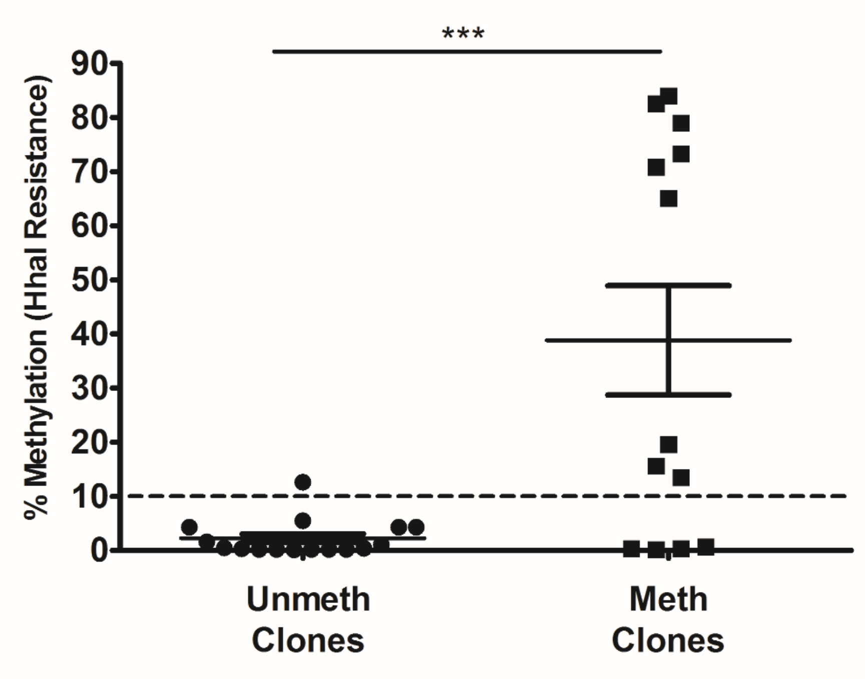

Supplemental Figure 5: MSRE-qPCR of HEK293T clonal lines repaired with either an unmethylated or methylated repair template. Cells were analyzed for HDR and methylation ~5 weeks post transfection. Shown here are clones that had at least 25% HDR (measured via SspI digest). n = 16 unmeth clones, 13 meth clones. Two-tailed t-test, p = 0.0004.

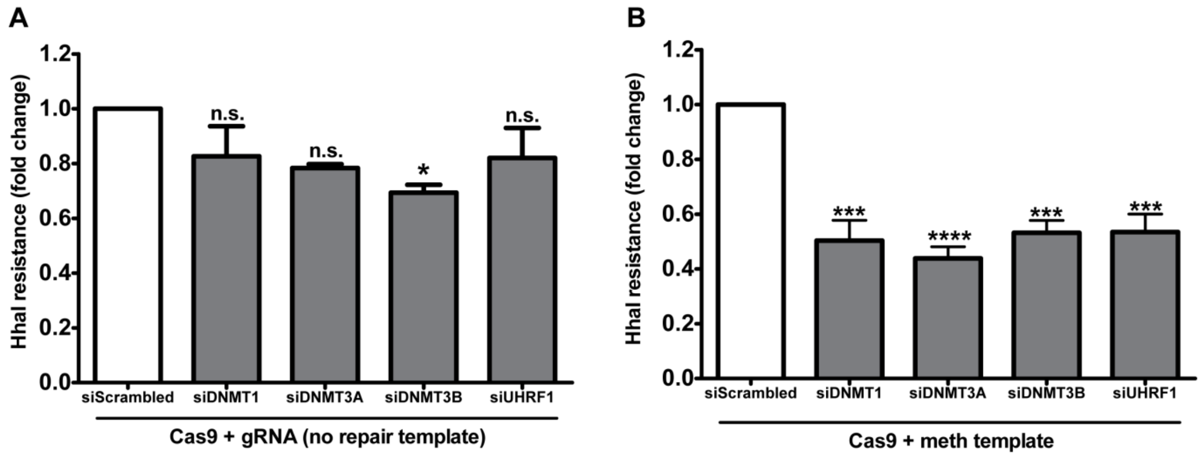

Supplemental Figure 6: Effect of DNMT knockdown on targeted methylation of *C9orf72*. siRNA was transfected 24 hours prior to transfection of either Cas9+gRNA and no repair template **(A)** or Cas9+gRNA and 1.4kb methylated template **(B)** into HEK293T cells. Cells were collected 4 days post transfection. Methylation was measured via MSRE-qPCR and normalized to scrambled control. n = 3 experiments; One-way ANOVA followed by Bonferroni post-hoc tests. *p < 0.05; *** p < 0.001; **** p < 0.0001.

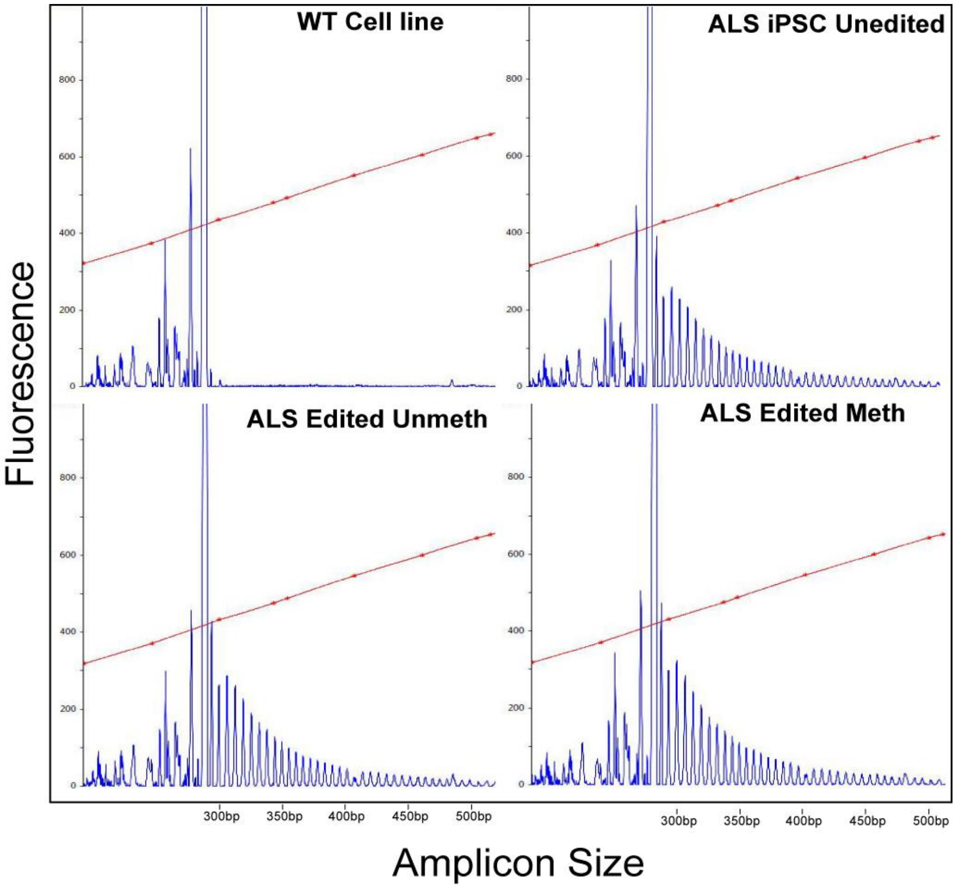

Supplemental Figure 7: Repeat Primed PCR of iPSCs derived from ALS patient with C9rof72 repeat expansion. Capillary electrophoresis traces from fluorescently labeled repeat primed PCR on unedited or edited clonal iPSC lines repaired with either the 1.4kb unmethylated dsDNA template or methylated dsDNA template.

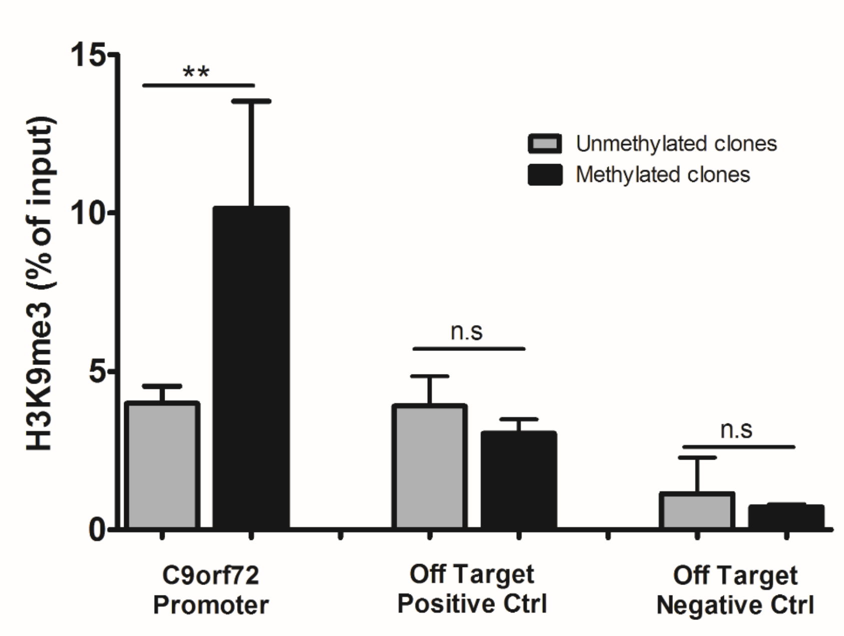

Supplemental Figure 8: H3K9me3 chromatin immunoprecipitation followed by qPCR of unmethylated and methylated clonal iPSCs. Off-target positive control is the *PAPC1* gene body, whereas the negative control is the *PAPC1* promoter region. n = 3 cell lines per group and 2 technical replicates. One-way ANOVA (p = 0.0001) followed by Bonferroni post-hoc tests between indicated groups. **p < 0. 01.

Table S1: Primers and gRNA sequences

Forward Primer Sequence (5'-3')	Reverse Primer Sequence (5'-3')	Assay
CAGTGTGAAAATCATGCTTGAGAGA	TTTGTGCTTGGTAGGCAGTG	DNA methylation analysis q-PCR; ChIP-qPCR
TGCTCTTTGAGAAAATTCATTGG	TTTGTGCTTGGTAGGCAGTG	DNA methylation analysis q-PCR (long templates)
TGGCACTATTAAGGATCTGAGGAG	GGAGAGAGGGTGGGAAAAAC	HDR analysis PCR (long templates)
TACCAGGGTTTGCAGTGGAG	GGAGAGAGGGTGGGAAAAAC	T7/HDR analysis (oligo templates)
GGAGTTGTTTTTTATTAGGGTTTGTAGT	TAAACCCACACCTACTCTTACTAAA	Bisulfite Cloning PCR
AGTTTTTTTTATTAAGGTTYGTATA	TTTCTTCTAATTAATCTTTATCAA	Bisulfite Cloning Primer Insert - WT
TTTAGTAGTTTTTGAATAATATTGG	TTTCTTCTAATTAATCTTTATCAA	Bisulfite Cloning Primer Insert - Mutant
CCCACTTCATAGAGTGTGTGTTG	TTCCATTCTCTCTGTGCCTTC	Total *C9orf72* mRNA (all variants)
CGGTGGCGAGTGGATATCTC	TGGGCAAAGAGTCGACATCA	*C9orf72* variant 2 mRNA
GCAAGAGCAGGTGTGGGTTT	TGGGCAAAGAGTCGACATCA	*C9orf72* variant 3 mRNA
6FAM-AGTCGCTAGAGGCGAAAGC	TACGCATCCCAGTTTGAGACG	*C9orf72* Repeat Expansion Detection
TACGCATCCCAGTTTGAGACGGGGGCCGGGGCCGGGGCCGGGG		*C9orf72* Repeat Expansion Detection
caccgTAGCGTGTGCGAACCTTAAT	aaacATTAAGGTTCGCACACGCTAc	*C9orf72* gRNA sequences (oligos for cloning)
caccgAGGTGAGTCCTAGGACGCTG	aaacCAGCGTCCTAGGACTCACCTc	*APP* gRNA sequences (oligos for cloning)
GATTTTTTTAGAGGAATAATATTGG	TTCTCTACATTAAAAACTTAAATTAAAATC	*APP* bisulfite cloning primer insert (mutant)

Table S2: Repair template sequences with PAM mutation highlighted

Name	Sequence	Length (bp)
Non-homologous template	CAGAAGAAGAAGGGCTCCCATCACATCAACCGGTGGCGCATTGCCACGAAGCAGGCCAATGGGGAGGACATCGATGTCACCTCCAATGACAAGTTGCT AGCGGTGGGCAACCACAAACCCACGAGGGCAGAGTGCTGCTTGCTGCTGGCCAGGCCCCTGCGTGGGCCCAAGCTGGACTCTGGCCACTCCCT	191
C9orf72 190bp ssOligo template	CTAGCGAAATGGGGCGGGGCAACTTGTCCTGTTCTTTTATCTTAAGACCCGCTCTGGAGGAGCGTTGGCGCAATAGCGTGTGCGAAC CTTAATATTGGAGGCTGCTGGATCTGGAGAAAGTGAAGACGATTTCGTGGTTTTGAATGGTTTTGTTTGTGCTTGGTAGGCAGTGGGCGCTCAACACATAATT	190
C9orf72 600bp dsDNA template	AGGCACCGCA [illegible] TTAATATTGGAG [illegible] CACTGTTAAG	621
C9orf72 1.4kb dsDNA template	CCCCGCCCCG [illegible] TTAATATTGGAG [illegible] CTCACCAGTT	1415
C9orf72 PCR-insert template	AACTGGTGAG [illegible] GAATAATATTGG [illegible] CACAGTACTC	1248
APP PCR-insert template	TGCTTAACAA [illegible] GAATAATATTGG [illegible] GGGGACGCGA	1058

Supplemental Methods:

T7 Endonuclease Assay and HDR digest Assay

A T7 endonuclease assay was used to measure mutation (indel +HDR) efficiency based on T7 digestion of mismatched DNA hybrids. 40ng of genomic DNA was amplified with 0.5U Q5 hotstart polymerase (New England Biolabs) in a reaction containing 0.2mM dNTP, 1X Q5 reaction buffer, 1X Q5 High GC enhancer, 0.25µM each primer (See Supplemental Table 1 for primers). 12ul of unpurified PCR product was hybridized with the following conditions: 95^0 5min; Cool to 85^0 at -2^0/sec (60% ramp rate); Cool to 25^0 at -0.1^0/sec (3% ramp rate). Hybridized products were then digested with 5U T7 Endonuclease I (New England Biolabs) for 30 minutes at 37^0. Digest products were run on 2% agarose gels and stained with ethidium bromide. Bands were quantified using GelAnalyzer 2010a software (http://www.gelanalyzer.com/index.html) using valley to valley background subtraction. For calculating T7 digest efficiency, we used the formula: % digest = 100 x (1 – (1- fraction cleaved)$^{1/2}$) as described previously(1).

Indel Analysis via sanger sequencing

40ng of genomic DNA was amplified with Q5 hotstart polymerase (New England Biolabs) in a reaction containing 0.2mM dNTP, 1X Q5 reaction buffer, 1X Q5 High GC enhancer, 0.25µM each primer (same as used for HDR analysis PCR (long templates), see Supplemental Table 1) and 0.5U Q5 hotstart enzyme. PCR conditions were as follows: 98° for 2 mins; 33 cycles of 98^0 10 sec, 64° 30 sec, 72° for 39 sec; final extension 72° for 2 mins. PCR products were purified using Qiagen PCR purification kit, eluted in 30ul EB buffer and ~100ng was Sanger sequenced using the inside primer: 5’-TACCAGGGTTTGCAGTGGAG-3’ using an ABI 3730 (Applied

Biosystems). Trace (.ab1) files were then uploaded to Synthego ICE web platform (https://ice.synthego.com/#/) to calculate the "Inference of CRISPR Edits" as described(2).

HhaI Digest Bisulfite Sequencing of CRISPR Edited DNA

10ug of genomic DNA was digested with 30U of HhaI (New England Biolabs) in 1X Cutsmart buffer (40ul reaction) for 16 hours at 37° C followed by 80° for 20 minutes to inactivate the enzyme. Digested DNA was purified using 1.8X Ampure XP beads (Beckman Coulter) to enrich for fragments >100bp and eluted in 20ul EB buffer. 2ug of digested DNA were then bisulfite converted using the Qiagen Epitect Kit and bisulfite amplicon cloning was carried out as described in the main text.

Chromatin Immunoprecipitation

Edited iPSC clonal lines were cross-linked with 1% formaldehyde (37^{0} C, 10 minutes) and quenched with 125μM glycine (37^{0} C, 10 minutes) in 10cm dishes. Cells were then washed with cold PBS once, then additional cold PBS was added and cells were scrapped into tubes and pelleted at 2,000rpm at 4^{0} C. PBS was removed and cells were lysed using cold buffer containing 0.5% SDS, 10mM EDTA, 50mM Tris/HCl, pH 8 + 1X protease inhibitors and chromatin from 2 million cells was transferred to Bioruptor tubes for sonication. Sonication was carried out on high power for 30 cycles (30 sec on, 30 sec off) using a Bioruptor 300 (Diagenode, Denville, NJ), and chromatin was sheared to ~200-500bp and analyzed by gel electrophoresis. Sheared chromatin was spun at 21,000g for 10 mins at 4^{0} C and supernatant was taken for immunoprecipitation. Chromatin was then diluted 5X using buffer containing 0.01% SDS, 1.1% TritonX, 1.2mM EDTA, 16.7mM Tris/HCl, and 167mM NaCl, pH 8. 1μg of

chromatin was incubated overnight at 4^0 C with 30ul of Protein A Dynabeads (Life Technologies) and 5µl of antibody against H3K9me3 (Cell Signaling #13969) or IgG. 10% of the input was set aside. Beads were washed with buffers of increasing stringency: Low salt: 0.1% SDS, 1% TritonX, 1.2mM EDTA, 16.7mM Tris/HCl pH 8, 150mM NaCl; high salt: 0.1% SDS, 1% TritonX, 1.2mM EDTA, 16.7mM Tris/HCl pH 8, 550mM NaCl; lithium chloride: 0.25M LiCl, 1% NP-40, 1% sodium deoxycholate, 1mM EDTA, 10mM Tris/HCl pH 8, followed by a wash with 1X TE buffer. Chromatin was eluted in buffer containing 1% SDS, 50mM Tris/HCl pH, 1mM EDTA. RNA and protein in the IP samples and inputs were digested using 80ug of RNAse A (Thermo Scientific, EN0531) and 60ug proteinase K (Promega, MC5005) for 16 hrs at 65 ^{0}C. DNA was purified using PCR purification kit (Qiagen) and eluted in 200µl EB Buffer (Qiagen). 4µl purified DNA was inputted into the same qPCR reaction as described in the methylation sensitive restriction enzyme digest quantitative PCR (MSRE-qPCR) assay. Primers for *C9orf72* were the same used for MSRE-qPCR (listed in Table S1). H3K9me3 positive control primers: Forward 5'-CCCAGCCACTGGGAAGCT-3'; Reverse 5'-TCACAGGCACAATCACAACACA-3'; Negative control primers: Forward 5'-TCCTCTCTGCTCTTTCCTCCTGTT-3'; Reverse 5'-TCCTGCACCCTCTACTTATACCC-3' The percentage of input was calculated as follows: $\Delta CT = CT\ (input) - CT\ (chromatin\ IP)$; % total input = $2^{\Delta CT} \times 10.0\%$.

Supplemental References

1. Miller,J.C., Holmes,M.C., Wang,J., Guschin,D.Y., Lee,Y.-L., Rupniewski,I., Beausejour,C.M., Waite,A.J., Wang,N.S., Kim,K.A., *et al.* (2007) An improved zinc-finger nuclease architecture for highly specific genome editing. *Nat. Biotechnol.*, **25**, 778–85.
2. Hsiau,T., Conant,D., Rossi,N., Maures,T., Waite,K., Yang,J., Joshi,S., Kelso,R., Holden,K., Enzmann,B.L., *et al.* (2019) Inference of CRISPR Edits from Sanger Trace Data. *bioRxiv*, 10.1101/251082.
